# Locoregional staging of breast cancer: contrast-enhanced mammography versus breast magnetic resonance imaging

**DOI:** 10.1007/s11547-024-01789-9

**Published:** 2024-03-21

**Authors:** Andrea Terzoni, Paola Basile, Anna Clelia Gambaro, Silvia Attanasio, Anna Maria Rampi, Marco Brambilla, Alessandro Carriero

**Affiliations:** 1 Scuola di Specializzazione Radiodiagnostica, University of Piemonte Orientale, Novara, Italy; 2 SCDU Radiodiagnostica Maggiore della Carità, Novara, Italy; 3 Health Physics Department, University Hospital, Novara, Italy

**Keywords:** Contrast-enhanced mammography, Breast cancer, Magnetic resonance imaging

## Abstract

**Purpose:**

Breast cancer diagnosis often involves assessing the locoregional spread of the disease through MRI, as multicentricity, multifocality and/or bilaterality are increasingly common. Contrast-enhanced mammography (CEM) is emerging as a potential alternative method. This study compares the performance of CEM and MRI in preoperative staging of women with confirmed breast carcinoma. Patients were also asked to fill in a satisfaction questionnaire to rate their comfort level with each investigation.

**Methods:**

From May 1st, 2021 to May 1st, 2022, we enrolled 70 women with confirmed breast carcinoma who were candidates for surgery. For pre-operative locoregional staging, all patients underwent CEM and MRI examination, which two radiologists evaluated blindly. We further investigated all suspicious locations for disease spread, identified by both CEM and MRI, with a second-look ultrasound (US) and eventual histological examination.

**Results:**

In our study cohort, MRI and CEM identified 114 and 102 areas of focal contrast enhancement, respectively. A true discrepancy between MRI and CEM occurred in 9 out of 70 patients examined. MRI reported 8 additional lesions that proved to be false positives on second-look US in 6 patients, while it identified 4 lesions that were not detected by CEM and were pathological (true positives) in 3 patients.

**Conclusions:**

CEM showed results comparable to MRI in the staging of breast cancer in our study population, with a high rate of patient acceptability.

## Introduction

Breast cancer is the most diagnosed malignant tumor and the leading cause of death among women [1, 2]. The evolution of surgical techniques and a more conservative approach [3–5], along with the detection of multi-focal, multicentric and/or bilateral mammary neoplasms [6], have changed the preoperative routine, which now requires an accurate locoregional staging of disease [7], in order to select the cases that, according to the immunophenotype assessment, can benefit from target therapy.

Currently, MRI with contrast medium is the gold standard for evaluating disease extension [8] in patients with confirmed breast cancer diagnosis, with diagnostic sensitivity and specificity values of 95% and 53%, respectively [9]. In this regard, a study on 1352 patients with breast cancer showed that performing an MRI before surgery changed the therapeutic/surgical pathway in 241 of them [10]. MRI has however some drawbacks: it should preferably be performed during the period from the seventh to the fourteenth day of the menstrual cycle, it is not always available on the territory, and it requires machines used in multiple diagnostic fields (1.5–3 T). This creates a shortage of MRI machines and long waiting times for patients who need them, not only for breast cancer diagnosis but also for other medical conditions. Moreover, it is an expensive investigation with intrinsic methodological limitations, unpopular among patients, especially if claustrophobic [11]. In this context, contrast-enhanced mammography (CEM) has opened up new scenarios and offered a valid alternative to MRI also in the pre-operative planning of patients with diagnosed breast cancer [12]. CEM has in fact numerous potential advantages as it is an easily accessible examination, easy to perform [13], with lower costs than MRI, potentially more available on the territory, and independent of menstrual cycle. It could therefore optimize economic and human resources as well as reduce waiting times [14]. The disadvantages of CEM compared to MRI is the use of ionizing radiation and the “topographic limitations”, especially for what concerns the detection of the lymph node involvement, while the risks related to the use of an intravenous contrast medium are shared with MRI.

The purpose of our study was to prospectively evaluate the diagnostic accuracy of CEM *vs* MRI for locoregional staging of patients with a diagnosed breast neoplasia, independently from the histological type. We also investigated patient preference for the two imaging methods.

## Materials and methods

### Ethics, study population, and study design

With prior authorisation from the Ethics Committee No. 118/20, we consecutively and prospectively enrolled 70 women with a breast cancer diagnosis confirmed by US-guided biopsy and candidates for surgery at the Radiodiagnostic Department of the Ospedale Maggiore della Carità in Novara and the University of Eastern Piedmont, from May 1st, 2021 to May 1st, 2022. All patients were informed about the objectives and methods of the tests, and their signed informed consent was obtained to perform the tests. For enrollment purposes, inclusion criteria were as follows:

- Women in pre-admission with already confirmed invasive breast cancer (BIRADS-6 according to ACR Birads 2013 edition, T1-2 and G 1/2/3 according to the Eltson-Ellis system);
- Age over 30 years;
- Negative history for adverse events related to the use of iodinated and gadolinium-based contrast agents;
- Normal renal function assessed in the last 3 months (documented by creatinine and glomerular filtration rate values).

Exclusion criteria were as follows:

- Women with breast implants;
- Pregnancy;
- Women under 30 years of age;
- Positive history for serious adverse events related to the use of iodinated and gadolinium;
- Altered renal function;
- Any other MRI contraindication that could interfere with the study.

All patients with confirmed breast cancer were subjected to CEM and MRI for preoperative locoregional staging within a period of 10 days, with no need for a precise order of execution between the two exams. All suspicious locations for disease spread identified both with CEM and MRI were investigated, after a second-look ultrasound (US), through US-guided histological examination.

According to our study protocol, CEM and MRI were evaluated blindly by two expert breast radiologists. In case of discrepancy between the two techniques, the radiologists discussed the case together in order to produce a single final report (after a US-second look and an eventual US-guided biopsy).

### Technique

#### CEM acquisition

CEM was performed with a Hologic Selenia Dimension digital mammography system. Before performing the CEM examination, a venous access was placed in the forearm, through which a low-osmolarity iodinated contrast agent (Iomeron 350) was administered in a single dose using an injector (Bracco Injeneering EmpowerCTA®) at an injection rate of 2–3 ml/sec, followed by washing with 20 ml of saline solution. The intravenous injection of the contrast medium was performed with the patient in a sitting position. The volume of contrast administered was equal to 1.5 ml/kg of body weight (with a maximum of 110 ml).

Starting from the pathological breast, there were acquired two mammographic projections with dual-energy (in order craniocaudal and medio-lateral oblique in tomosynthesis), 1 min after the end of the injection. The examination was completed with late acquisitions in craniocaudal and medio-lateral oblique projections in 2D, acquired in the same order of the previous ones, starting from the seventh minute after administration of the contrast medium. The late projections were acquired in 2D in order to prevent an additional radiation dose for the patient.

### MRI acquisition

All examinations were performed with a 3 T Philips superconducting magnet and dedicated coils, with the patient in a prone position. A standard protocol was performed, including a T2-weighted sequence (axial and sagittal), a T1-weighted gradient echo sequence (axial) before and after infusion of paramagnetic contrast medium, DWI/ADC sequences (b values from 0 to 850) and subtracted sequences (6 acquisitions sequentially obtained). Gadolinium (Gadovist, Bayern) was used at a dose of 1 ml/kg. The contrast medium was infused using a Spectris Solaris EP injector at an infusion rate of 2 ml/min followed by physiological infusion, for an average examination time of about 25 min.

### Image analysis

Two experienced breast radiologists evaluated the CEM and MRI exams blindly and independently. Subsequently, the two radiologists compared their findings in order to prepare a single final structured report (using ACR Birads lexicon according to ACR Birads 2013 edition) to be made available to the patient and the surgeon for correct therapeutic planning.

The readers, who were aware of the presence and location of a primary heteroplastic lesion, were asked, blindly, to define: i) the presence of any additional areas of pathological contrast enhancement that could raise suspicion of multifocality and/or multicentricity and/or bilaterality; and ii) the total number and topographic location, for each patient, of the contrast enhancement areas detected by the individual methods. Successively, the information provided by the two readers was compared to evaluate concordance or discordance. Suspicious lesions with a discrepancy were subjected to a second-look US and, eventually, to histological investigation.

Any adverse events due to the injection of the contrast medium during each examination were recorded on a supplementary patient-related card.

At the end of both investigations, the patients completed a questionnaire to comparatively evaluate their satisfaction with the two exams. The questionnaire included the following questions: 1) Which of the two exams caused you greater anxiety? 2) During the execution of the exams, in which of the two exams did you feel more comfortable? 3) In the event of a future check-up, which of the two methods would you prefer?

### Statistical analysis

Concordance analysis was performed on a patient basis and on a lesion basis. Data were summarized as experimental percentages together with 95% confidence intervals (CI). Lastly we asked every patient to fill in a satisfaction questionnaire to rate their comfort level with each investigation (expressed as a percentage).

## Results

In our study, a total of 70 patients with a histological diagnosis of breast cancer were enrolled. From the analysis of their medical histories, we found that: the average age was 58.4 years (with a range of 33–81 years); 51/70 women were menopausal, while 19/70 still had regular menstrual cycles. Among the patients, 60/70 had had at least one pregnancy and, of these, 49 had breastfed. A family history of breast carcinoma was reported by 14/70 patients.

The patients reported the following recent clinical history:

- 37 patients (53%) had presented themselves spontaneously for check-ups after finding nodules through self-examination:
- 23 patients (32%) had come for check-ups following recalls for second-level investigations as part of their regional screening program;
- 10 patients (14%) had received an accidental diagnosis of breast carcinoma following routine breast clinical-diagnostic checks.

### Histological data

From the analysis of the histopathological reports of the target lesions analyzed, a clear prevalence of the ductal histotype 78.57% (55/70) compared to the lobular one 17.14% (12/70) was found. In one case (1.43%), an undifferentiated carcinoma was detected, whereas in two cases (2.86%) it was found a mucinous carcinoma.

Most of these lesions, at the time of biopsy, already had an infiltrating pattern (61 cases), while in nine cases a carcinoma in situ was uncovered. The totality of the patients underwent a US examination also extended to the axillary cavities as per standard practice and, of the 34 patients who underwent a biopsy also in this location, 22 were diagnosed with lymph node metastatic localization.

Tables 1 and 2 reported the 114 and 102 areas of focal contrast enhancement identified on MRI and CEM, stratified by the number of synchronous lesions seen on a single patient, respectively. In particular, all the primary lesions proved to have a corresponding finding in both CEM and MRI examination, in terms of localisation and pathological imaging features. In Table 3 the concordance between the two classifications (MRI and CEM) was reported, on the basis of the US findings.

**Table 1:** Lesions identified on MRI

MRI synchronous lesions	Frequency
1	39
2	20
3	9
4	2

**Table 2:** Lesions identified on CEM

CEM total lesions	Frequency
1	49
2	15
3	5
4	2

**Table 3:** Concordance of results between the methods on a lesion basis

	CEM +	CEM −
RM +	102	12
RM −	0	0

**Fig. 1:**
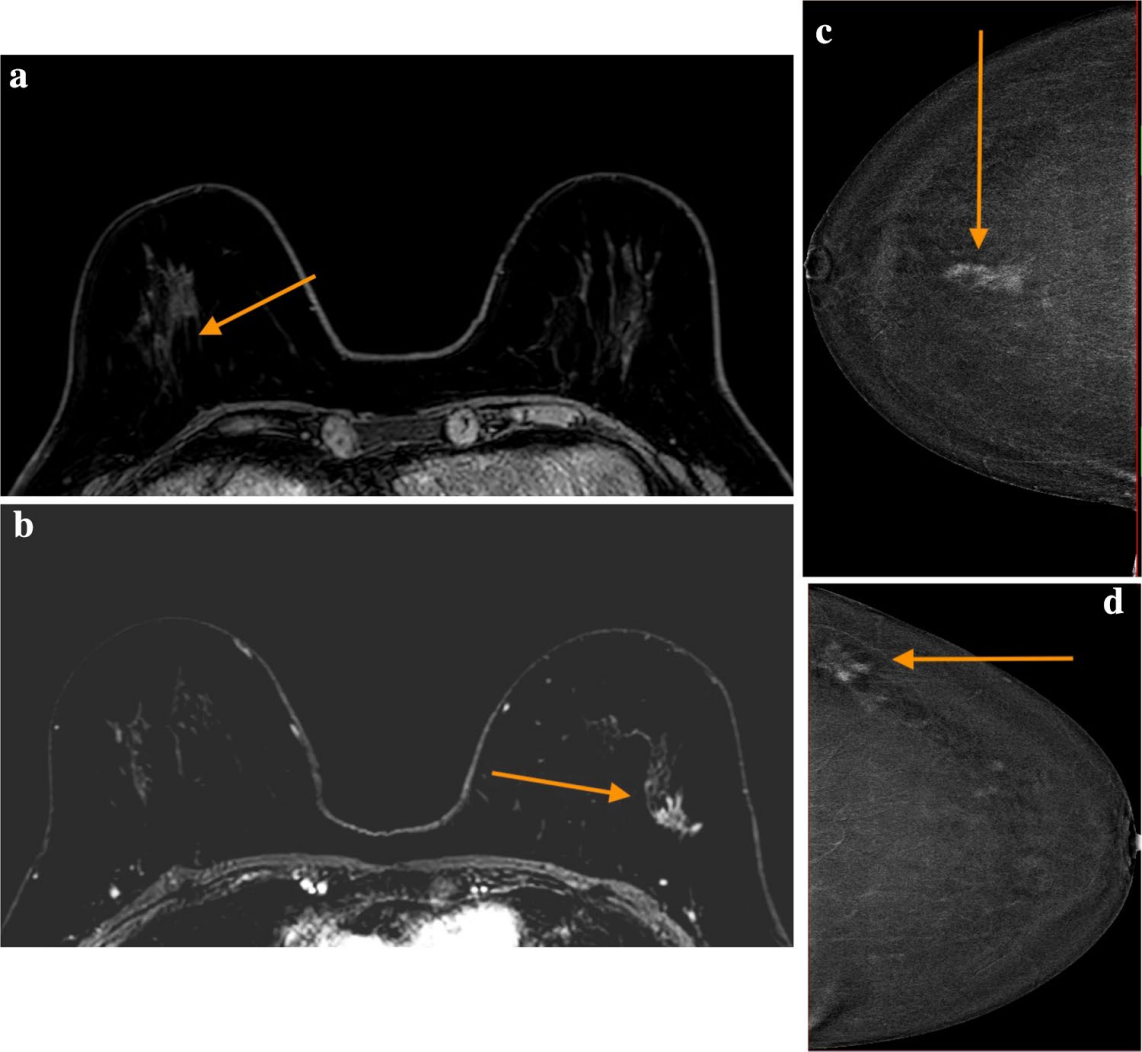
**a** Concordance in patient 1 MRI, right side lesion [1]. **b** Concordance in patient 1 MRI, left side lesion [2]. **c** Concordance in patient 1 CEM, right side lesion [1]. **d** Concordance in patient 1 CEM, left side lesion [2]

**Fig. 2:**
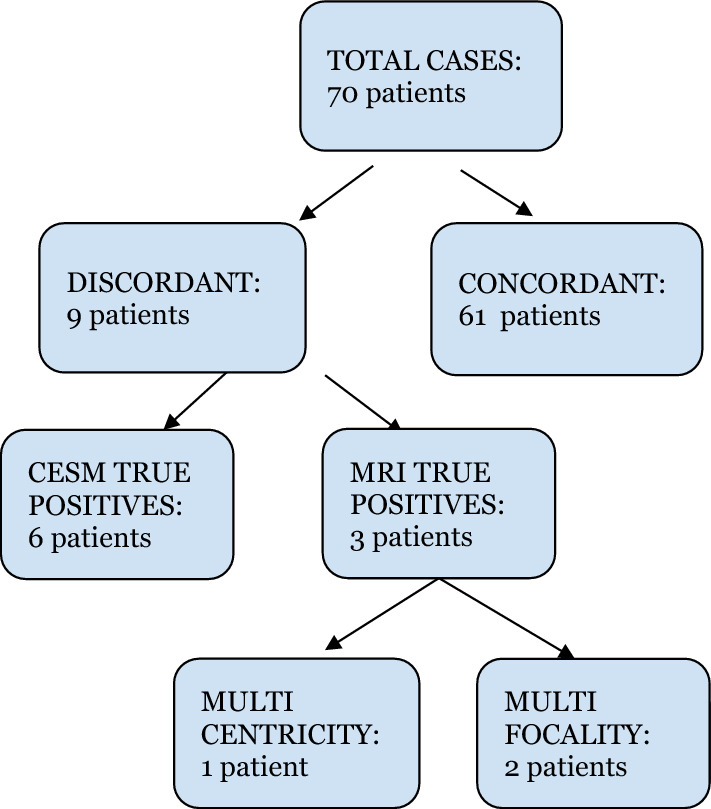
Concordance of results between the methods on a patient basis

On a lesion based analysis, out of the 114 lesions seen at MRI, CEM correctly identified 102 lesions (89.5%; 95%CI 83.9–95.1)(Table 3). On a patient basis, MRI and CEM agreed in recognizing malignant lesions in 61 out of 70 cases (87.1%; 95%CI 79.3–95.0%) (Fig. 1a–c, 1d), thus leading to a true discrepancy between MRI and CEM in 9 out of 70 women examined. In particular, in 6 out of 9 patients, MRI identified a total of 8 additional lesions that, on US second-look, turned out to be “false positives” (Table 4), since they did not have a US corresponding finding and consequently they were not biopsied. Whereas in 3 out of 9 patients MRI detected 4 lesions that were not identified by CEM, but proved to be pathological at the following US-guided biopsy. The results of the concordance between the two methods are shown in Fig. 2.

**Table 4:** US second-look of the discrepant lesions

	US + (true positive)	US − (false positive)
RM +	4	8

**Fig. 3:**
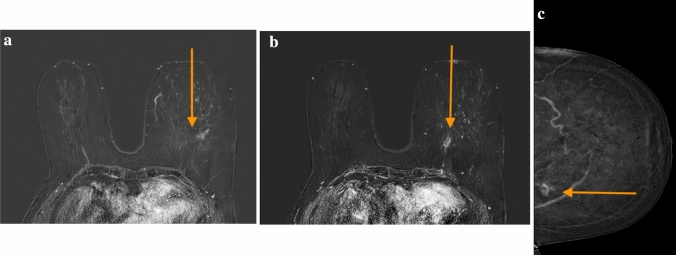
**a** Discordance in patient 2 MRI, left side first lesion [1]. **b** Discordance in patient 2 MRI, left side second lesion [2]. **c** Discordance in patient 2 CEM, left side unique lesion

With regard to these last three cases, two patients had lobular breast carcinoma, while the third was diagnosed with invasive ductal breast carcinoma, histologically concordant with the primary lesion. Specifically:

- Case 1 had a multifocal invasive ductal carcinoma with a maximum size of 12 mm that was detected by MRI as additional lesion (Fig. 3a–c);
- Case 2 displayed a multifocal lobular carcinoma with a maximum diameter of 10 mm, identified as above with MRI;
- Case 3 had a multicentric lobular carcinoma, diagnosed by detecting two neoplastic focal formations on MRI in different breast quadrants, measuring 7 mm and 9 mm, respectively.

In all three cases examined, the finding of additional lesions on MRI did not, however, lead to any change in surgical strategy, as a mastectomy had already been planned based on the focal lesions found on first-level exams.

In our study protocol, all suspicious locations for disease spread, additional to the index lesion, identified at CEM and/or MRI, were re-evaluated using US second-look as our gold standard and all the times that they had an US equivalent, it was investigated using US-guided biopsy to confirm its neoplastic nature.

The comparison between CEM and MRI revealed some differences in the diagnosis of bilaterality, multifocality, and multicentricity of breast cancer. From the comparative evaluation of CEM and MRI it emerged that:

- MRI found bilaterality in 10/70 cases (14%; 95% CI 8–24%) versus 6/70 cases (9%; 95% CI 4–18%) for CEM. After subsequent US and/or histological investigation, the gold standard confirmed bilaterality in 6 cases. In particular, the cases identified by CEM turned out to be true positives, while MRI identified 4 false positives.
- MRI diagnosed multifocality in 17/70 cases (24%; 95% CI 16–36%) versus 13/70 (19%; 95% CI 11–29%) for CEM. After subsequent US and/or histological investigation, the gold standard confirmed multifocality in 15 patients. In particular, there were 2 false negative cases recognized by CEM and 2 false positive cases uncovered by MRI.
- MRI identified multicentricity in 16/70 cases (23%; 95% CI 15–34%) versus 10/70 (14%; 95% CI 8–24%) for CEM. After subsequent US and/or histological investigation, the integrated gold standard confirmed multicentricity in 11 patients, with 5 false positives recognized by MRI and only one false negative case by CEM.

As for the safety of the examination, no adverse reaction to the contrast medium was recorded in a population of patients who declared no allergies.

### Satisfaction results

In the satisfaction questionnaire, the patients answered three questions about their experience with CEM and MRI (Table 5). The questions and summarized answers were as follows:

1. N1) Which of the two exams caused you greater anxiety?
  All patients (70/70–100%) reported that the greatest state of anxiety was present before undergoing MRI.
2. N2) During the execution of the exams, in which of the two exams did you feel more comfortable?
  Only in 2 cases was the answer in favor of MRI, while 68 patients felt more comfortable during CESM.
3. N3) In the event of a future check-up, which of the two methods would you prefer?
  In all cases examined (70/70–100%), patients stated that in cases of future check-ups they would have preferred to undergo CESM.

**Table 5:** Answers of the 70 patients examined related to the survey of the two exams

Question	CESM	MRI
N1	0 (0%)	70 (100%)
N2	68 (97.2%)	2 (2.8%)
N3	70 (100%)	0 (0%)

## Discussion

MRI is recognized as the gold standard for the diagnosis of breast cancer, particularly for preoperative staging aimed to define the locoregional and/or bilateral extension of breast carcinoma [8]. In the last few years, CEM has emerged as a diagnostic tool that can rival MRI in its ability to detect and assess breast lesions [15, 16]. Both methods, however, work on the same principle: they use contrast to highlight the abnormal blood vessels that feed the diseased areas. Thus, the aim of this work was to evaluate, in our experience, the potential of CEM versus MRI in preoperative staging of women with histologically confirmed breast carcinoma.

In our study, we found that CEM and MRI detected the same number of lesions in 61/70 patients examined (about 87%). In three of the remaining nine cases (5%), only MRI could detect 4 areas of enhancement that were then found to be expression of disease spread, instead in the other six cases (8%), CEM did not reveal 8 suspicious lesions, which had instead been detected by MRI, that turned out to be false positives on second-level investigations, confirming the greater specificity of CEM versus MRI [17].

According to our study protocol, CEM and MRI were evaluated blindly by two expert breast radiologists. In case of disagreement between the two techniques, the radiologists discussed the case together in order to produce a single final report (after a US-second look and an eventual US-guided biopsy).

In 2013, Jochelson et al. [17] conducted a study of CEM and MRI in women with confirmed breast cancer. Their results showed that both CEM and MRI had similar target lesion detection rates (96%), which were much higher than those of conventional mammography (81%). Furthermore, CEM was less sensitive than MRI in finding multiple lesions (56% vs. 88%), but more specific (2 vs. 13 false positives).

Compared to our study, Lee-Felker et al. [16] compared the diagnostic performance of CEM *vs* MRI in identifying the target tumor lesion and any additional focal points in women with recent breast cancer diagnosis, also using US and US-guided biopsy as second-line methods. From their experience, regarding the 120 total lesions found in 52 women, it emerged that CEM obtained a sensitivity value similar to that of MRI (94% vs. 99%), a higher PPV value (93% vs. 60%), and a lower index of false positives (5 cases vs. 45). In particular, CEM also identified 11/11 confirmed secondary lesions, while MRI identified 10/11.

A 2018 study by Kim et al. [15] challenged some of these data. They compared CEM and MRI in a population of 84 women and found similar sensitivity values in identifying the target lesion (92.9% for CEM and 95.2% for MRI), but no significant differences in identifying additional ipsilateral or contralateral lesions. They also reported that the method did not influence the surgical strategy; for both CEM and MRI, the surgical approach was changed for 26 and 25 out of 84 patients respectively based on the diagnostic findings. Another study by Lee-Felker et al. [16] supported these data. They also compared CEM and MRI in women with recent breast cancer diagnosis, using US and US-guided biopsy as second-line methods. They found that CEM had similar sensitivity (94% vs. 99%), higher positive predictive value (PPV) (93% vs. 60%), and lower false positives (5 vs. 45 cases) than MRI in detecting the target tumor lesion and any additional focal points. Out of the 120 total lesions found in 52 women, CEM identified all 11 confirmed secondary lesions, while MRI missed one.

### Topographic limitations

CEM has some topographic limitations that may affect its diagnostic accuracy. Some lesions may not be visible on CEM because they are located outside the acquisition plane. These include: the axillary cavity (and thus pathological lymph nodes) [18], the submammary fold and all deep anatomical regions, which may not be properly investigated depending on the patient’s physical constitution and compliance as well as to the technicians’ expertise [19]. In our experience, CEM detected suspected lymphadenopathies in 20 cases versus 22 for MRI. However, both methods require further diagnostic investigations and/or intraoperative confirmation because lymph node contrast enhancement is a non-specific finding. Nevertheless, these topographic limitations must be overcome by integrating CEM with a second-look US [18].

Apart from this limitation, CEM offers many benefits such as ease of execution, reduced time, low costs and greater patient compliance, making this examination widely available to the population. One of the advantages of contrast mammography is its significantly shorter duration (about 10 min) compared to that of MRI, which requires the patient to maintain the prone position for 25 min for correct acquisition. Moreover, CEM images are easier to read for radiologists who are familiar with traditional mammography [20]. In addition, the radiation dose of contrast mammography, although higher than traditional mammography, remains below that recommended by European and British guidelines [21]. As for the safety of the examination, no adverse reaction to the contrast medium was recorded in a population of patients who declared no allergies.

## Conclusions

CEM is increasingly establishing itself as an alternative method to MRI in the preoperative staging of patients with breast carcinoma, as documented by the growing interest in this investigation in the literature. In particular, a concordance emerges in the judgment of non-inferiority and equality between CEM and MRI in the identification of breast carcinoma, as well as a superiority of CEM in terms of specificity, PPV, ease of execution of the examination, as well as accessibility and acceptability by the patient. Furthermore, compared to MRI, the cost–benefit ratio is not negligible: in fact, from a cost point of view it has been calculated that the average cost of a CEM is $200 compared to the average cost of an MRI which is $800 [22]. A further advantage of CEM is that it would reduce the overall waiting list for diagnostic imaging. Indeed, mammography equipment is more widely available on the territory than superconducting magnets, which would allow MRI to be used for other types of pathologies. Therefore, we recommend CEM as a safe and effective alternative imaging approach to MRI for preoperative staging of breast carcinoma.
